# Hepatitis B virus prevalence, immunization and immune response in people living with HIV/AIDS in Istanbul, Turkey: a 21-year data analysis

**DOI:** 10.4314/ahs.v21i4.16

**Published:** 2021-12

**Authors:** Esra Zerdali, Inci Yilmaz Nakir, Serkan Surme, Mustafa Yildirim

**Affiliations:** 1 Haseki Education Research Hospital, Infectious Diseases and Clinical Microbiology, İstanbul, Turkey

**Keywords:** HIV, Hepatitis B, prevalence

## Abstract

**Objective:**

We aimed to determine Hepatitis B virus (HBV) prevalence, immune status, and the prevalence of antibody response in people living with HIV/AIDS (PLWHA) in Istanbul, Turkey.

**Methods:**

The study includes PLWHA aged 18 years and older who were followed-up for at least 6 months from 1997 to 2018.

**Results:**

Of the 653 patients with PLWHA, 99 (15.2%) were both antiHBc-IgG and antiHBs positive, 120 (18.3%) were antiHBc-IgG positive/antiHBs negative. HBsAg was positive in 40 (6.1%) patients. HBsAg positive coinfection (≤40 years 4.6% vs. >40 years 21.7%, p<0.001) and antiHBc-IgG positivity/antiHBs negativity (≤40 years 14.0% vs. >40 years 26.5, p<0.001) were higher in PLWHA older than 40 years. The prevalence of HIV/HBV coinfection reached a peak level of 22.2% in 2004, and it decreased to 3.3% in 2018. The prevalence of immunization before HIV diagnosis was low (15.6%). The prevalence of antibody response (anti-HBs>10 IU/L) after immunization for HBV was 50%. A higher protective response was associated with CD4+≥350 cell/mm3 (59.3%, p=0.014).

**Conclusion:**

HBV coexistence in PLWHA remains an imperatively important problem. The most conclusive methods in solving this problem are to prevent transmission by immunization and control measures. Also, HBV screening should in no manner be neglected in PLWHA.

## Introduction

According to the World Health Organization, 38 million people are living with HIV/AIDS (PLWHA), and HIV and Hepatitis B virus (HBV) coinfection occurs in 7.4 % of individuals 1,2. Although HBV and HIV have similar transmission routes, such as blood, sexual intercourse, and mother-to-child transmission, HBV is approximately 100-fold more contagious than HIV 3. HBV, on the other hand, is more likely to cause acute hepatitis in PLWHA, and these individuals are at increased risk for developing chronic hepatitis^4^.

There is an increased risk of mortality and morbidity in HIV/HBV coinfected individuals due to the negative effects of HBV on the liver. In PLWHA, active HBV replication increases liver damage, accelerates progression to fibrosis, and facilitates the development of liver cirrhosis and hepatocellular cancer (HCC) 5. HIV is also associated with increased occult HBV and low spontaneous clearance rates 6,7,8. It is also known that HBV can cause immune reconstitution inflammatory syndrome in individuals living with HIV 9. Because of these, treatment approaches for PLWHA are planned according to the presence of HBV coinfection 5,10,11. As a result, it is vital to determine HBV coinfection in PLWHA as it shapes therapeutic approaches and prognostic evaluation.

When studies conducted in the world and especially in Turkey are evaluated, it is seen that there are not enough contemporary epidemiological studies about HIV and HBV coexistence. In this study, we aimed to determine HBV prevalence, immune status, and the prevalence of antibody response after immunization in PLWHA in Istanbul, Turkey. Therefore, we will contribute to epidemiological data in Turkey by determining the frequency of HBV, immune status and antibody formation prevalence after HBV vaccination in PLWHA who admitted to our hospital.

## Methods

The study included PLWHA at the age of 18 years and older who were followed-up for at least 6 months from 1997 to 2018 in Infectious Diseases and Clinical Microbiology Department of Haseki Training and Research Hospital. Seven hundred-seventy six patients met eligibility criteria. Of whom, 123 were excluded because of insufficient data. A total of 653 PLWHA were included in this study. HBsAg, anti-HBcIgG, anti-HBs, HBeAg, delta antibody positivity status, HBV DNA and alanine aminotransferase (ALT) levels were retrospectively examined and recorded in the data recording form. Also, the antibody responses of the patients who were vaccinated for hepatitis B were evaluated.

HBsAg loss has observed more frequently in PLWHA compared to the general population, and occult hepatitis B has frequently encountered in repeated examinations^7^,^8^. Although acute HBV is more likely to develop into a chronic infection in PLWHA than in HBV monoinfected patients, conventional serological markers may be insufficient to detect chronic HBV. The use of HBV active antiretroviral treatment (ART) may cause seroconversion that manifest as loss of HBsAg. Because of that, individuals with AntiHBc IgG positive/AntiHBs negative were also evaluated. Patients with chronic hepatitis B were divided into four groups in terms of the presence of HBV e antigen (HBeAg) and chronic hepatitis/chronic infection according to the updated version in the European Association for the Study of the Liver (EASL) 2017 Clinical Practice Guidelines of hepatitis B virus infection 11. These four groups were defined as HBeAg(+) chronic infection, HBeAg(+) hepatititis, HBeAg(-) chronic infection, and HBeAg(-) hepatitis according to the positivity of HBeAg, as well as the presence of HBV DNA (reference range: 31.6-20,000,000 IU/ml, the clinically relevant cut-off values: >107 IU/ml, 104–107 IU/ml, <2000 IU/ml, and >2000 IU/ml respectively) and ALT levels (reference range:0–41 IU/L, the clinically relevant cut-off value: 41 IU/L). Additionally, occult HBV infection was defined as HBV DNA detection in serum in patients who tested negative for HBsAg.

Each dose of HBV vaccine was 20 mcg of HBsAg. The minimum interval between the first and second doses of the HBV vaccine was four weeks. The minimum interval between the first and third doses of the vaccine was six months. The threshold for protective anti-HBs positivity was a quantitative anti-HBs titer of greater than 10 IU/L. Antibody response was evaluated 1–2 months after the last dose of the vaccine.Additionally, we evaluated factors (including age, gender, the duration of ART, viral load, and CD4+ T lymphocyte count at the time of vaccination) associated with the protective antibody response to HBV vaccine.

Median, minimum and maximum values were presented for continuous data without normal distribution. Chi-square test was used to analyze categorical data. Kolmogorov-Smirnov test was used to test for a normal distribution. Independent Samples t-test and Mann-Whitney U test were used for comparison of continuous variables between two independent groups. A p value below 0.05 within the 95% Confidence Interval was considered statistically significant. Statistical analyses were conducted using SPSS version 22.0 (tatistical Package for the Social Sciences, Chicago, IL, USA). This study was carried out by following relevant laws, guidelines and the World Medical Association, Helsinki Declaration, Ethical Principles for Medical Research Involving Human Subjects standards. Ethics committee approval was received from the Ministry of Health University Istanbul Training and Research Hospital Clinical Research Ethics Committee (Approval no: 1561 date: 07.12.2018).

## Results

There were 653 PLWHA included in the study. Of whom, 120 (18.3%) were antiHBc-IgG positive/anti-HBs negative (HBsAg positive or negative), 99 (15.2%) were both antiHBc-IgG and antiHBs positive, 40 (6.1%) were HBsAg positive.

The mean age of the patients with antiHBc-IgG positive/antiHBs negative (n=120) was 40.3 ± 10.9 (17–74) years and 106 (88.3%) of them were male. The age range with the highest frequency of antiHBc-IgG positive/antiHBs negative patients was 41–50. The mean age of HBsAg positive coinfected patients (n=40) was 37.4 ±9.5 (17–59) years. The age range with the highest frequency of HBsAg positive coinfected patients was 31–40. The distribution of patients according to age and gender is given in Table 1.

**Table 1:** The distribution of patients with HIV/HBV coexistance according to age and gender

	Anti HBc IgG (+)/AntiHBs (-)			
	HBsAg (+/-)		HBsAg (+)	
	n	%	n	%
**Number of patients**	120	100	40	100
**Gender**				
**Female**	14	11.7	3	7.5
**Male**	106	88.3	37	92.5
**Age**				
**Mean ±sd**	40.3 ±10.9		37.4 ±9.5	
**Median** **(minimum-maximum)**	40.5 17–74		37 17–59	
**18–30**	27	22.5	11	27.5
**31–40**	33	27.5	14	35.0
**41–50**	41	34.2	13	32.5
**51–60**	15	12.5	2	5.0
**>60**	4	3.3	0	0

While there was no relationship between HBV coinfection and gender in PLWHA (p=0.665), a statistically significant relationship was found between HBV coinfections and age (p<0.001). Moreover, HBsAg positive coinfection (≤40 years 4.6% and >40 years 21.7%, p<0.001) and antiHBc-IgG positivity/antiHBs negativity prevalence (≤40 years 14.0% and >40 years 26.5% p<0.001) were found to be significantly higher in the individuals older than 40 years.

The prevalance of antiHBc-IgG positivity was 33.5% (n=219), while the HBsAg positive coinfection prevalence was 6.1% (n=40). HBsAg positivity was 18.3% (40/219) among patients with antiHBc-IgG positive. Occult HBV infection was detected in three patients (0.5%), while only one patient (0.2%) had Delta hepatitis. The distribution of patients with hepatitis B coinfection according to the EASL 2017 Clinical Practice Guideline and presence of antiHBc-IgG is presented in Table 2.

**Table 2:** The distribution of patients with hepatitis B coinfection according to the EASL 2017 Clinical Practice Guideline^11^ and presence of anti-HBV IgG

	n	%
**Number of patients**	653	100
**AntiHBc-IgG (-) (n=434)**		
**AntiHBs (-)**	332	50.8
**AntiHBs (+)**	102	15.6
**AntiHBc-IgG (+) (n=219)**	219	33.5
**HBsAg (-) AntiHBs (+)**	99	15.2
**HBsAg (-) AntiHBs (-)**	80	12.2
**HBsAg (+) AntiHBs (-)**	40	6.1
**HBeAg(+) Chronic Infection**	2	0.3
**HBeAg(+) Hepatitis**	2	0.3
**HBeAg(-) Chronic Infection**	15	2.3
**HBeAg(-) Hepatitis**	19	2.9

There were 102 patients (15.6%) vaccinated before HIV diagnosis. The status of hepatitis B vaccination prevalence and the occurrence of adequate antibody levels after vaccination in PLWHA are presented in Table 3. Age (p=0.586), gender (p=0.844), duration of ART (p=0.383), and viral load (p=0.773) at the time of vaccination were not associated with a poor antibody response. HBV vaccine response was significantly lower in PLWHA vaccinated at CD4+ T lymphocyte count <350 cell/ mm3 than in PLWHA vaccinated at CD4+ T lymphocyte count ≥350 cell/ mm^3^ (n=44, 41.9% vs. n=54, 59.3%, p=0.014) (Table 4).

**Table 3:** Hepatitis B vaccination status in people living with HIV/AIDS

			Antibody response	
	n	%	n	%
**Total number**	653	100	-	-
**Vaccinated before HIV** **diagnosis**	102	15.6	-	-
**Vaccinated after HIV** **diagnosis**	196	30.0	98	50.0
**Isolated AntiHBc IgG (+)**	29	4.4	13	44.8
**AntiHBc IgG (-)**	167	25.6	85	50.9

**Table 4:** Factors associated with a protective antibody response to HBV vaccine

	Protective antibody response (anti-HBs>10 IU/L)		

Total (n=196)	Presence (n=98)	Absence (n=98)	p
**Age (years)**			0.586
Mean ±sd	37.9 ±11.0	36.9 ±10.7	
**Gender**			0.844
Male n (%)	83 (84.7%)	82 (83.7%)	
Female n (%)	15 (15.3%)	16 (16.3%)	
**Duration of ART before vaccination (weeks)**			0.383
<8 weeks n (%)	55 (56.1%)	61 (62.2%)	
≥8 weeks n (%)	43 (43.9%)	37 (37.8%)	
**Viral load at the time of vaccination (IU/ml)**			0.773
<100,000 n (%)	41 (41.8%)	43 (43.9%)	
.100,000 n (%)	57 (58.2%)	55 (56.1%)	
**CD4^+^ T lymphocyte count at the time of vaccination (cell/ mm^3^)**			**0.014**
<350 n (%)	44 (44.9%)	61 (62.2%)	
≥350 n (%)	54 (55.1%)	37 (37.8%)	

ART= Antiretroviral treatment

**Figure 1:**
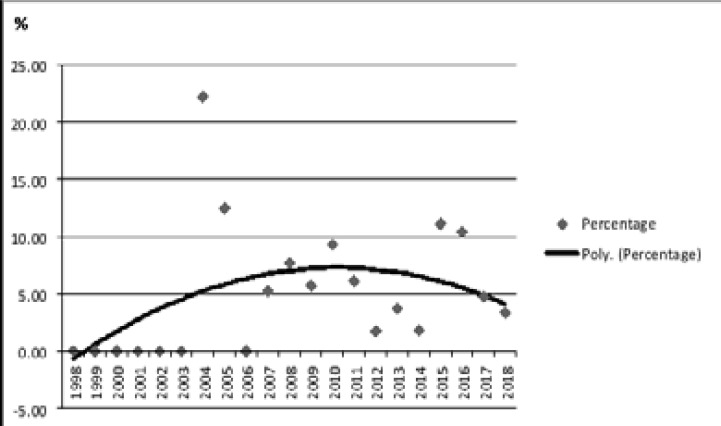
Hepatitis B prevalence in people living with HIV/AIDS by years

The prevalence of HIV/HBV coinfection reached a peak level of 22.2% in 2004, and it decreased to 3.3% in 2018 (Figure 1).

## Discussion

In this study, we demonstrated a high prevalence of exposure to HBV and reduced antibody response to immunization in PLWHA. HBsAg positive coinfection (≤40 years 4.6% vs. >40 years 21.7%, p<0.001) and antiHBc-IgG positivity/antiHBs negativity prevalence (≤40 years 14.0% vs. >40 years 26.5, p<0.001) were found to be higher in the individuals older than 40 years. We also showed that while the prevalence of HIV/HBV coinfection reached a peak level of 22.2% in 2004, and it decreased to 3.3% in 2018.

Turkey is accepted as the moderate epidemic of hepatitis B by the World Health Organization. In the TURHEP study, which included 5460 patients representing the general population conducted by Tozun et al., HBsAg, antiHBs and antiHBc were found in 4.0%, 31.9% and 30.6%, respectively^12^. In our study, the prevalences of HBsAg, antiHBs, and antiHBc-IgG were determined as 6.1%, 30.8% and 33.5%for HBsAg, antiHBs, and anti-HBc-IgG, respectively. As detected in our study, there was no significant difference in HBV serology in PLWHA compared to the general population of Turkey. In the study of Tozun et al., many factors such as living in the Southeastern region, male gender, low educational status and history of dental operation were identified as risk factors for HBsAg positivity, but age (≥50) was identified as the only risk factor for anti-HCV positivity. Also, in the same study, HBeAg positivity was lower among HBsAg positive individuals (4.1%) compared to our data, while the frequency of hepatitis D (2.8%) was similar with our data 12.

Frequency of hepatitis B differs according to geographical regions in the world as well as in Turkey 11, 12,13. Moreover, studies are showing that the frequency of hepatitis B decreases over the years. Emekdas et al.11, in a study involving approximately 6 million blood donors, observed that HBsAg positivity prevalence decreased from 5.2% to 2.1%. Similar rate (2.6%) was shown in the study of Dilek et al. 14. In the study of Tanrıverdi et al., which included 35,295 pregnant women, HBsAg positivity prevalence decreased from 1.4% in 2013 to 0.8% in 2016, and antiHBs positivity prevalence increased from 25.4% to 30.2% 15. It can be thought that the decrease in the frequency of HBV seen in Turkey is achieved by increasing infection control measures, vaccination policy and providing free healthcare services. Thanks to similar applications, it can be suggested that coinfection prevalence in our centre decreased from 22.2% to 3.3% in 2018. With ageing, antibody levels may decrease and protection provided by vaccine may decrease. On the other hand, we think that the age factor is not an independent factor that increases the risk of coinfection in our study, especially because the HBV vaccination has been made a compulsory measure for young people.

In the study of Karaosmanoğlu et al.^16^, they included 71 patients living with HIV between 1998–2008, HBsAg, antiHBs and antiHBc-IgG positivity were found as 4.2%, 11.1% and 33.8%, respectively. In another study of Karaosmanoğlu et al.^17^, covering the years 2006–2011, antiHBc positivity among individuals living with HIV was found to be 19.1%. Also in the same study, occult hepatitis B was found to be 7.5% (n = 3/40) among isolated antiHBc positive individuals. In a study conducted in Italy, the HIV/HBV coinfection ratio was found to be 4.1%. In the same study, HIV/HBV coinfection showed worse clinical and immunological features than HIV/HCV coinfection^18^. In the study conducted by Silva et al.^19^ in Brazil, the HIV/HBV coinfection prevalence was 3.1%. Although there was no statistically significant difference in CD4+ T lymphocyte ratio compared to those who were monoinfected with HIV, a significantly increased CD4+ T lymphocyte levels in the HIV/HBV coinfected group after 24 months of antiretroviral therapy was detected. While occult hepatitis B was not evaluated in the study of Silva et al. 19, in another study conducted in Brazil, the prevalence of occult hepatitis B was found 14% (n = 6/43) in PLWHA with antiHBc-IgG positive^20^. Since occult HBV infection may play a role in HBV reactivation, transmission of HBV, HCC development, and liver disease progression, screening occult HBV is is an important issue in the management of PLWHA 21. In our study, the prevalence of occult hepatitis B was found 2.5% (n = 3/120) in PLWHA with antiHBc-IgG positive. In the study of Hofer et al., on the other hand, at least one HBV DNA positivity was detected in 89.5% of patients in repeated HBV DNA measurements in isolated HIV positive individuals with isolated anti-HBc-IgG positivity 7.

In the study conducted by Matthews et al. In Sub-Saharan Africa, HBsAg positivity prevalence was found to be 7% among 950 women living with HIV/AIDS, but no hepatitis Delta was detected. 22. In our study, the frequency of hepatitis D was found to be 0.2% among PLWHA and 2.5% among HIV/HBV coinfected individuals. In a study conducted in Turkey, the frequency of hepatitis D was found to be 8.8% among patients with chronic HBV 23. We think that the significant difference observed in the frequency of Delta hepatitis is because Hepatitis D, which is considered endemic in Turkey, is affected by geographical differences.

Another issue in our study was that HBV vaccination prevalence (n=298/653, 45.6%) and post-vaccination antibody response (n=98/196, 50.0%) in PLWHA were low. This weak response may be due to three single-dose (20 µg intramuscular at day 0, 1 month and<=>6 months) administration of HBV vaccine. Additionally, our results showed that HBV vaccine response was significantly lower in PLWHA vaccinated at CD4+ T lymphocyte count <350 cell/mm^3^. This result was consistent with other studies 24, 25. Fonseca et al. showed that CD4+ T lymphocyte counts ≥350 cells/mm3 were associated with a higher seroconversion rate in PLWHA vaccinated with double dose 25. In a randomized controlled study, Launey et al., reported that four intramuscular double doses or four intradermal low doses significantly increased antibody response^26^. Therefore, if antiHBs level is lower than <10 IU/L, re-vaccination with double-dose should be considered. Re-vaccination may be deferred until CD4+ T lymphocyte count reaches ≥350 cells/mm^3^. Although antibody levels are lower than the general population, HBV vaccination is important to prevent the development of coinfecton, to prevent acute and chronic liver damage, and also to reduce treatment costs 27. Besides, the vaccine prevents the transmission of HBV to other individuals in the community 28.

Strengths of our study are that the study was conducted in a centre that has been following PLWHA for years and has a high number of patients. The limitations of our study are that various factors, except for age and gender, such as transmission routes, socio-economic status, and chronic liver inflammation and fibrosis by liver biopsy were not evaluated. Besides, the fact that the study was conducted in a single-centre is another limitation.

## Conclusion

Antibody response to immunization is poor, while the prevalence of HBV among PLWHA has been high. Therefore, the presence of HBV coexistence in PLWHA remains an imperatively important problem. The most conclusive methods in solving this problems are to mitigate transmission by immunization and control measures. Also, HBV screening should in no manner be neglected in PLWHA. We recommend that all PLWHA in Turkey should be routinely screened for HBsAg, anti-HBC-IgG and antiHBs before being vaccinated.
